# A Decade of HIV Preexposure Prophylaxis (PrEP): Overcoming Access Barriers in the United States Through Expanded Delivery

**DOI:** 10.1177/00333549231208487

**Published:** 2023-11-30

**Authors:** Kristopher J. Jackson, Sandra I. McCoy, Douglas A.E. White

**Affiliations:** 1 Center for AIDS Prevention Studies, University of California, San Francisco, San Francisco, CA, USA; 2 Division of Epidemiology, University of California, Berkeley, Berkeley, CA, USA; 3 Highland Hospital, Alameda Health System, Oakland, CA, USA

**Keywords:** HIV, preexposure prophylaxis, PrEP

During the past decade, preexposure prophylaxis (PrEP) has become the centerpiece of HIV prevention in the United States^1^ and in some parts of the world.^2^ There were approximately 1 million active PrEP users worldwide as of 2019.^2^ While PrEP uptake has increased over time, recent estimates suggest that only 25% of people in the United States who might benefit from PrEP have been prescribed 1 of the 3 antiviral medications approved for use as PrEP by the US Food and Drug Administration (FDA).^3^ More disheartening, rates of PrEP use and PrEP awareness among Black/African American and Hispanic/Latino/a/x men who have sex with men (MSM) as well as Black/African American and Hispanic/Latino/a/x transgender and cisgender women are disproportionately low, while new cases of HIV are disproportionately high in these groups.^4,5^ A goal central to the Ending the HIV Epidemic initiative in the United States is to increase PrEP use to at least 50% of the estimated 1.2 million people in the United States at risk for acquiring HIV by 2030.^3,6^ Given the disparities in PrEP prescribing and PrEP access in the United States, meeting this goal requires changing how clinicians, public health experts, and policy makers view HIV prevention and PrEP.

## PrEP Access: Opportunities and Pitfalls

Section 2713 of the Affordable Care Act (ACA) addresses private health plan coverage of preventive health services relevant to PrEP coverage.^7^ Following the 2019 US Preventive Services Task Force recommendation statement and evidence report granting PrEP an “A rating” as an HIV prevention strategy, most private US health plans were—pursuant to Section 2713 of the ACA—required to cover PrEP with no out-of-pocket cost incurred by plan beneficiaries.^8,9^ Unfortunately, Section 2713 does not help people in the United States who are at risk for HIV who lack health insurance coverage for prescriptions. The future of such mandatory health insurance coverage of PrEP under Section 2713 remains uncertain in the wake of *Braidwood Management v Becerra*, a legal challenge to the preventive services provision of the ACA currently being argued in the US District Court in the Northern District of Texas.^10^

Health insurance coverage is integral to PrEP’s success as an HIV prevention strategy in the United States, but it is merely one piece of the PrEP access conundrum. Even with widespread health insurance coverage, only a fraction of PrEP-eligible people in the United States receive PrEP. At the patient level, not all people with health insurance seek preventive medical care despite engaging in behaviors that increase their risk of acquiring HIV. Furthermore, many people may not know about PrEP and/or may not feel comfortable sharing their sexual histories or drug use behaviors with a health care provider. Conversely, some health care providers may lack the time or willingness to obtain such patient histories and/or may not feel comfortable prescribing PrEP. To achieve Ending the HIV Epidemic 2030 goals, we posit 3 interrelated processes that are essential for PrEP uptake in the United States: person-centered PrEP regimens, PrEP education for health care providers, and evidence-based implementation approaches that increase PrEP access (Figure).

**Figure.**
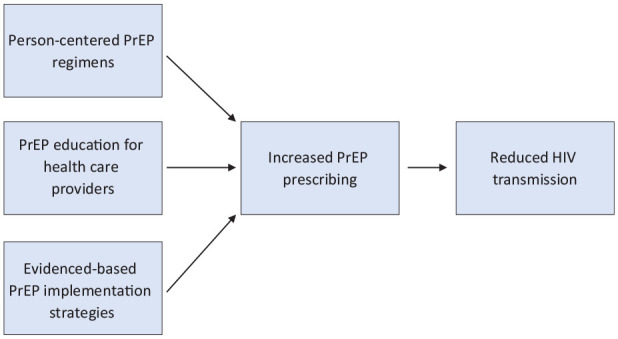
Interrelated processes that are essential for preexposure prophylaxis (PrEP) uptake in the United States.

### Person-Centered PrEP Regimens

Emtricitabine/tenofovir disoproxil fumarate (TDF/FTC) was the first formulation approved for use as PrEP in the United States.^1^ Taken as prescribed, this daily oral regimen can be nearly 100% effective in preventing HIV transmission among MSM^11-14^ and is an effective HIV prevention strategy among those who engage in behaviors associated with HIV acquisition.^15,16^ In 2019, emtricitabine/tenofovir alafenamide was the second combination agent that FDA approved for PrEP use.^17^ For some people, however, a daily oral PrEP regimen may not be feasible or practical. The newest antiretroviral addition to the global HIV prevention toolkit, cabotegravir, is a long-acting injectable administered bimonthly, which may prove especially valuable for patients whose adherence to a daily oral medication is challenging.^18^

While event-driven (ie, on-demand) oral PrEP is not currently FDA approved, trials exploring the efficacy of event-driven oral PrEP dosing strategies appear promising, and the 2021 PrEP Clinical Practice Guidelines from the Centers for Disease Control and Prevention support this practice.^19^ A 2 + 1 + 1 regimen (eg, taking a TDF/FTC dose 2-24 hours prior to sexual intercourse and a daily dose for 2 days thereafter) may be an effective HIV prevention strategy among MSM^20^ and is described in the PrEP Clinical Practice Guidelines.^19^ Event-driven PrEP addresses the practical reality that sexual risk is dynamic, many people can anticipate sexual activity, and adherence to daily oral therapy can be difficult for some people. Some practitioners and health departments in the United States offer guidance for event-driven PrEP as an alternative to daily or injectable PrEP in selected populations at high risk for HIV acquisition.^19,20^

Recently updated guidance supports same-day PrEP prescribing.^19^ In patients for whom immediate or near-term HIV and renal function testing is possible, initiating same-day PrEP may reduce barriers to access and increase patient uptake by not requiring the patient to undergo such testing or an additional clinical consultation before initiating PrEP.

More PrEP options mean individuals at risk for HIV could eventually select from multiple effective prevention options tailored to personal need and preference. HIV prevention, much like HIV care, is not a one-size-fits-all endeavor; a successful prevention strategy for one person may be impractical for another. With a pipeline of new PrEP formulations on the horizon and multiple strategies on hand, health care providers must remain abreast of available and emerging PrEP strategies that can make PrEP more convenient and less dependent on daily or monthly adherence.

### PrEP Education for Health Care Providers

#### Combating the “purview paradox.”

While PrEP uptake has increased over time in the United States, questions remain about which clinicians are responsible for prescribing PrEP in clinical practice. This phenomenon, known as the “purview paradox,” refers to ambiguity surrounding which health care providers should or could screen patients for PrEP eligibility and prescribe PrEP.^21^ Infectious disease practitioners are likely most experienced with HIV treatment and prevention, but these providers are in limited supply; it is unreasonable to expect the estimated 9000 practicing infectious disease physicians in the United States^22^ to provide PrEP to the approximately 1.2 million people in the United States who might benefit from this HIV prevention method.^3^

Viewed through a lens of disease prevention, PrEP might be well suited to the primary care setting. Currently, more than 120 000 primary care or internal medicine physicians are active in the United States, and disease prevention is a cornerstone of primary care practice.^22^ While PrEP prescribing has been steadily increasing over time in the primary care setting,^23^ some primary care providers are reluctant to discuss or offer PrEP to their patients. Reasons for this reluctance include a PrEP knowledge deficit, unease prescribing a new or unfamiliar medicine, discussing HIV risk with patients, perceived cost, and competing clinical priorities.^24-26^ While PrEP is currently far from universally available in the primary care setting in the United States, some primary care practices have made a concerted effort to offer PrEP to patients.^26^ Some health systems have integrated PrEP clinics and developed a streamlined referral process to these clinics into their model of care delivery.^27,28^ However, opportunities may exist for expanded PrEP access beyond primary care. Some intrepid health care providers are exploring alternative models for PrEP delivery in women’s health clinics,^29^ pharmacies, emergency departments (EDs),^30^ and outpatient substance use treatment centers.^31^

#### Risk versus benefit: combating compensatory risk

A growing body of evidence suggests that health care providers in the United States could be reluctant to prescribe PrEP because of personally held beliefs pertaining to PrEP, in particular the notion that offering their patients PrEP might encourage patients to engage in risky behaviors.^21,32^ The notion that people alter their behavior or make choices in response to perceived risk is known as “compensatory risk.”^33,34^ Risk compensation theory posits that people increase risk taking if offered an intervention that mitigates perceived risk.^33^ Thus, risk compensation theory might suggest that offering patients PrEP could encourage sexual risk-taking behavior given the mitigated risk of HIV transmission. Multiple randomized PrEP efficacy trials, however, found no self-reported increases in sexual risk-taking among study participants.^35,36^ A recent retrospective study of self-reported sexual behaviors among MSM found that PrEP use was associated with lower rates of condom use but was not associated with an increased number of sexual partners or substance use.^37^ Recent studies of MSM and transgender women have suggested that rates of bacterial sexually transmitted infections (STIs) are higher among PrEP users than among non–PrEP users, although other studies report that PrEP users are at no greater risk for STI transmission after PrEP initiation.^38-42^ Given these inconsistent findings, it is unclear how the mitigated risk of HIV offered by PrEP may contribute to rising rates of STIs in the United States. Emerging data suggest that the quarterly STI testing required of PrEP users may, over time, result in stabilization or declining of STI rates.^43^

The concept of risk compensation is not unique to PrEP. Health care providers routinely recommend harm-reduction services and prescribe treatments, medications, or lifestyle modifications to patients for health conditions or behaviors that could result in compensatory risk. Historic examples include the introduction of oral contraceptives in the 1960s or school-based condom distribution in the 1990s.^44,45^

Health care providers objecting to the provision of PrEP and/or limiting access to PrEP jeopardize the health and well-being of the communities they serve. Health care provider bias against PrEP must be addressed via institutional policy change to support practices such as universal PrEP eligibility screening and comprehensive sexual health history taking; health care provider education is necessary to ensure clinicians are equipped to have effective conversations with patients about these sensitive topics in a culturally competent manner—particularly given the intersectional stigma and racism affecting racial and ethnic and sexual or gender minority populations who are disproportionately affected by HIV. Continued investment in programs and interventions aimed at increasing PrEP uptake among Black/African American and Hispanic/Latino/a/x populations in the United States is vital to ending the HIV epidemic.

### Evidence-Based Implementation Approaches

#### PrEP prescribing in inclusive, culturally competent, person-centered environments

Fostering dialogue about sensitive, often stigmatized behaviors requires the provision of health care in an environment where patients feel comfortable discussing their sexual behavior(s) and other personal situations that might increase their risk for HIV transmission (eg, intravenous drug use, sex work, food insecurity, homelessness). Such an environment is particularly important for people with multiple intersecting marginalized identities, such as people in racial and ethnic minority groups who are also in sexual and gender minority groups, who routinely experience intersectional stigma at the health care provider and institutional levels when seeking routine medical care.^46,47^

Specialty health clinics for sexual and gender minority populations are typically viewed as safe spaces because they implement culturally competent primary and specialty care to the sexual and gender minority community members they serve.^46^ Many of these specialty clinics use an integrated care delivery model that offers patients combination HIV prevention services, care coordination and counseling, and peer support. While LGBTQIA+ community trust is essential to the delivery of sexual health and HIV prevention services in this population, not all people at risk for HIV choose to receive health care in these environments, nor do all people have access to such care. For example, MSM who are reluctant to disclose their sexuality or people who do not identify as MSM despite having sexual encounters with male-identifying people may be unlikely to frequent these clinics for fear of being “outed.” Restricting, either intentionally or unintentionally, the provision of PrEP to these clinical environments might risk limiting access to PrEP. Ensuring that all people at risk for HIV have access to PrEP means offering PrEP in diverse clinical environments, including those frequented by cisgender, heterosexual people and specialty practices serving subgroups such as LGBTQIA+ community members and people who inject drugs.

### Telehealth and TelePrEP

The COVID-19 pandemic jettisoned telehealth into the lives of health care consumers and providers. At the height of the pandemic, virtual care was nearly the only mechanism for receiving routine health care services in some settings. Increased institutional support and infrastructure to support telehealth visits, relaxed licensing requirements to provide telehealth services across state lines, and changes to reimbursement policies motivated health care providers to adopt telehealth.^48^ Just as telehealth has become an accepted mode of care delivery for many primary care and subspecialty practices, telehealth has made PrEP available to some patients from the comfort of their home.

Several internet-based providers prescribe PrEP via telehealth.^49,50^ Unlike patients who attend in-person PrEP visits, where HIV/STI testing is performed on-site, telehealth patients are given the option to visit a local laboratory or are sent a test kit to self-test for HIV and STIs. Patients repeat this process quarterly while taking PrEP in accordance with current clinical practice guidelines.^19^ Some PrEP-specific telehealth services, also referred to as “telePrEP,” mail PrEP to their patients in discreet packaging, eliminating the need to visit a pharmacy.

Like all implementation strategies, telePrEP has limitations. Telehealth providers do not have contracts with all health insurance providers. As a result, patients do not have equal access to this care delivery model. TelePrEP practices might elect to see uninsured patients on a fee-exempt or reduced, sliding-scale fee schedule, but the cost of medication and quarterly HIV testing remain barriers to PrEP access. Also notable, some populations (eg, racial and ethnic minority groups, transgender and gender-nonconforming people) are disproportionately unhoused or experiencing housing instability in the United States. Such populations may lack access to the technology necessary to conduct a telehealth visit.^51^

### PrEP Access via Community-Based Pharmacies

Approximately 90% of people in the United States live within 5 miles of at least 1 pharmacy.^52^ This is why, in part, retail pharmacies have emerged as venues for preventive care. Recognizing the need for increased PrEP access, as of 2021, legislation has been passed or introduced in more than 11 states, allowing pharmacists to dispense PrEP to patients—covered by third-party prescription coverage, if applicable—without an outside prescription.^53^ Signed into law in California in October 2019, Senate Bill 159 allows California pharmacists to dispense postexposure prophylaxis and a 60-day supply of PrEP to patients without a prescription.^54^

Differentiated PrEP delivery via community pharmacies could expand PrEP access to people who might not know about or who lack access to PrEP. However, barriers exist to initiating PrEP in the pharmacy setting, such as staffing, resources, and time required to screen patients for PrEP eligibility (eg, perform HIV testing), particularly because these services are time consuming, may be ineligible for reimbursement, and/or may not generate revenue. In some communities, no referral pathway to a prescriber may exist beyond the 60-day supply furnished by a pharmacist, heightening concerns about patient support, retention on PrEP, and prospects for future “prevention effective” use, a concept that aligns PrEP adherence with an individual’s risk of HIV while recognizing that adherence to or demand for PrEP may be periodic and associated with periods of increased or decreased risk of HIV acquisition.^55^

### PrEP . . . in the ED?

The ED has emerged as an important venue for disease screening and prevention initiatives in some US health care facilities, particularly because many people in the United States lack a primary care provider and, as of 2021, 1 in 10 people living in the United States lacked health insurance coverage.^56^ With respect to HIV, some US EDs have adopted an opt-out approach to HIV screening.^57,58^ Opt-out HIV screening has proven to be an effective strategy for diagnosing HIV infections in ED populations that serve patients who may lack access to routine health care services or who would not otherwise seek out HIV screening.^58^ In EDs with HIV screening and linkage-to-care programs, PrEP screening and delivery is a logical programmatic expansion. Several US EDs have conducted trials of PrEP initiatives^30^ and/or predictive algorithms/clinical decision support tools to identify patients who might benefit from PrEP.^59,60^

Prescribing PrEP in the ED has several implementation challenges. Emergency medicine providers’ knowledge of and familiarity with PrEP is one such limiting factor. Other factors limiting the ability to provide PrEP include the lack of efficient or reliable PrEP screening tools, the lack of requisite laboratory data, and the lack of reliable, established referral networks for patient follow-up.

Most EDs have infrastructure for contacting patients with abnormal diagnostic test results (ie, positive STI test results) and managing after-care referrals. Leveraging this infrastructure to connect patients to PrEP could make the ED a viable setting to reach a population that may not have health insurance or access to routine medical care and may not otherwise be offered PrEP. Conversely, discharging ED patients with an established HIV risk with a prescription for PrEP without a reliable follow-up plan may limit the feasibility of PrEP prescribing in this setting. However, emerging literature suggests that patient navigators—sometimes referred to as health care coordinators or care coordinators—are valuable assets to increasing PrEP uptake in ED and non-ED settings. These professionals provide patients with PrEP education, facilitate linkage to PrEP services, and could offer adherence and follow-up support to people who initiated PrEP in the ED.^61,62^

## Conclusion

PrEP is an effective HIV prevention strategy that currently reaches only a subset of people in the United States who might benefit from it. Disparities persist among populations disproportionately affected by HIV, with reduced access to health care services and reduced access to PrEP because of a constellation of personal and structural factors. HIV prevention is a dynamic field with evolving HIV prevention methods and additional PrEP formulations and PrEP delivery models are under way; person-centered PrEP formulations and strategies, such as event-driven PrEP or injectable PrEP, may further mitigate some individual-level factors such as adherence among PrEP users. Institutional and structural-level factors such as telePrEP, expanded venues for PrEP initiation, health care provider education, and other innovative approaches to PrEP delivery could improve PrEP access in the United States and should be the focus of future research.

## References

[1] CDC statement on FDA approval of drug for HIV prevention. Press release. Centers for Disease Control and Prevention; July 16, 2012. Accessed October 30, 2022. https://www.cdc.gov/nchhstp/newsroom/2012/fda-approvesdrugstatement.html

[2] Schaefer R Schmidt HMA Ravasi G , et al. Adoption of guidelines on and use of oral pre-exposure prophylaxis: a global summary and forecasting study. Lancet HIV. 2021;8(8):e502-e510. doi:10.1016/s2352-3018(21)00127-2 PMC8332196 34265283

[3] Centers for Disease Control and Prevention. PrEP for HIV prevention in the U.S. Published November 23, 2021. Accessed October 30, 2022. https://www.cdc.gov/nchhstp/newsroom/fact-sheets/hiv/PrEP-for-hiv-prevention-in-the-US-factsheet.html

[4] Kanny D Jeffries WL IV Chapin-Bardales J , et al. Racial/ethnic disparities in HIV preexposure prophylaxis among men who have sex with men—23 urban areas, 2017. MMWR Morb Mortal Wkly Rep. 2019;68(37):801-806. doi:10.15585/mmwr.mm6837a2 PMC6755820 31536484

[5] Centers for Disease Control and Prevention. Diagnoses of HIV infection in the United States and dependent areas, 2018: gay, bisexual, and other men who have sex with men. Published May 7, 2020. Accessed October 30, 2022. https://www.cdc.gov/hiv/library/reports/hiv-surveillance/vol-31/content/msm.html#race

[6] Centers for Disease Control and Prevention. Ending the HIV epidemic in the U.S. (EHE): prevent. Published June 13, 2022. Accessed October 30, 2022. https://www.cdc.gov/endhiv/prevent.html

[7] Kaiser Family Foundation. Preventive services covered by private health plans under the Affordable Care Act. Published May 15, 2023. Accessed October 30, 2022. https://www.kff.org/health-reform/fact-sheet/preventive-services-covered-by-private-health-plans

[8] US Preventive Services Task Force. Prevention of acquisition of HIV (HIV) infection: preexposure prophylaxis. Published June 11, 2019. Accessed September 8, 2023. https://www.uspreventiveservicestaskforce.org/uspstf/recommendation/prevention-of-human-immunodeficiency-virus-hiv-infection-pre-exposure-prophylaxis-june-2019

[9] Keith K . New guidance on PrEP: support services must be covered without cost-sharing. Health Affairs. Published July 28, 2021. Accessed June 10, 2023. http://healthaffairs.org/do/10.1377/forefront.20210728.333084

[10] *Braidwood Management Inc v Xavier Becerra* (4:20-CV-00283), District Court N.D. Texas (2020). Published August 7, 2023. Accessed October 26, 2023. https://litigationtracker.law.georgetown.edu/wp-content/uploads/2023/04/Braidwood_20230807_BRIEF-of-Braidwood-Management-et-al.pdf

[11] Grant RM Anderson PL McMahan V , et al. Uptake of pre-exposure prophylaxis, sexual practices, and HIV incidence in men and transgender women who have sex with men: a cohort study. Lancet Infect Dis. 2014;14(9):820-829. doi:10.1016/s1473-3099(14)70847-3 25065857 PMC6107918

[12] Liu AY Cohen SE Vittinghoff E , et al. Preexposure prophylaxis for HIV infection integrated with municipal- and community-based sexual health services. JAMA Intern Med. 2016;176(1):75-84. doi:10.1001/jamainternmed.2015.4683 26571482 PMC5042323

[13] McCormack S Dunn DT Desai M , et al. Pre-exposure prophylaxis to prevent the acquisition of HIV-1 infection (PROUD): effectiveness results from the pilot phase of a pragmatic open-label randomised trial. Lancet. 2016;387(10013):53-60. doi:10.1016/s0140-6736(15)00056-2 26364263 PMC4700047

[14] Volk JE Marcus JL Phengrasamy T , et al. No new HIV infections with increasing use of HIV preexposure prophylaxis in a clinical practice setting. Clin Infect Dis. 2015;61(10):1601-1603. doi:10.1093/cid/civ778 26334052 PMC4809999

[15] Choopanya K Martin M Suntharasamai P , et al. Antiretroviral prophylaxis for HIV infection in injecting drug users in Bangkok, Thailand (the Bangkok Tenofovir Study): a randomised, double-blind, placebo-controlled phase 3 trial. Lancet. 2013;381(9883):2083-2090. doi:10.1016/S0140-6736(13)61127-7 23769234

[16] Martin M Vanichseni S Suntharasamai P , et al. The impact of adherence to preexposure prophylaxis on the risk of HIV infection among people who inject drugs. AIDS. 2015;29(7):819-824. doi:10.1097/QAD.0000000000000613 25985403

[17] US Food and Drug Administration. FDA approves second drug to prevent HIV infection as part of ongoing efforts to end the HIV epidemic. Press release. October 3, 2019. Accessed September 8, 2023. https://www.fda.gov/news-events/press-announcements/fda-approves-second-drug-prevent-hiv-infection-part-ongoing-efforts-end-hiv-epidemic

[18] US Food and Drug Administration. FDA approves first injectable treatment for HIV pre-exposure prevention. Press release. December 20, 2021. Accessed September 8, 2023. https://www.fda.gov/news-events/press-announcements/fda-approves-first-injectable-treatment-hiv-pre-exposure-prevention

[19] Centers for Disease Control and Prevention. Preexposure prophylaxis for the prevention of HIV infection in the United States—2021 update: a clinical practice guideline. Published 2021. Accessed October 30, 2022. https://www.cdc.gov/hiv/pdf/risk/prep/cdc-hiv-prep-guidelines-2021.pdf

[20] Molina JM Ghosn J Assoumou L , et al. Daily and on-demand HIV pre-exposure prophylaxis with emtricitabine and tenofovir disoproxil (ANRS PREVENIR): a prospective observational cohort study. Lancet HIV. 2022;9(8):e554-e562. doi:10.1016/s2352-3018(22)00133-3 35772417

[21] Krakower D Ware N Mitty JA Maloney K Mayer KH . HIV providers’ perceived barriers and facilitators to implementing pre-exposure prophylaxis in care settings: a qualitative study. AIDS Behav. 2014;18(9):1712-1721. doi:10.1007/s10461-014-0839-3 24965676 PMC4127184

[22] American Association of Medical Colleges. 2020 Physician specialty data report: executive summary. 2021. Accessed June 10, 2023. https://www.aamc.org/media/50476/download?attachment

[23] Zhu W Huang YLA Kourtis AP Hoover KW . Trends in the number and characteristics of HIV pre-exposure prophylaxis providers in the United States, 2014-2019. J Acquir Immune Defic Syndr. 2021;88(3):282-289. doi:10.1097/QAI.0000000000002774 34651603 PMC8568068

[24] Henny KD Duke CC Geter A , et al. HIV-related training and correlates of knowledge, HIV screening and prescribing of nPEP and PrEP among primary care providers in Southeast United States, 2017. AIDS Behav. 2019;23(11):2926-2935. doi:10.1007/s10461-019-02545-1 31172333 PMC6803031

[25] Turner L Roepke A Wardell E Teitelman AM . Do you PrEP? A review of primary care provider knowledge of PrEP and attitudes on prescribing PrEP. J Assoc Nurses AIDS Care. 2018;29(1):83-92. doi:10.1016/j.jana.2017.11.002 29274655 PMC7653672

[26] Wilson K Bleasdale J Przybyla SM . Provider–patient communication on pre-exposure prophylaxis (PrEP) for HIV prevention: an exploration of healthcare provider challenges. Health Commun. 2021;36(13):1677-1686. doi:10.1080/10410236.2020.1787927 32633137 PMC10844925

[27] Konstantinovsky M . Can PrEP prevent HIV infection? One Medical. Published November 14, 2014. Accessed December 15, 2022. https://www.onemedical.com/blog/newsworthy/prep-hiv-prevention

[28] Kaiser Permanente. HIV care and prevention. 2022. Accessed December 15, 2022. https://thrive.kaiserpermanente.org/care-near-you/northern-california/sanfrancisco/departments/hiv-care-and-prevention

[29] American College of Obstetricians and Gynecologists. Preexposure prophylaxis for the prevention of human immunodeficiency virus. June 2022. Accessed October 30, 2022. https://www.acog.org/clinical/clinical-guidance/practice-advisory/articles/2022/06/preexposure-prophylaxis-for-the-prevention-of-human-immunodeficiency-virus

[30] Gormley MA Nagy TR Moschella P Lu Z Rodriguez J Roth P . HIV pre-exposure prophylaxis in the emergency department: a systematic review. Ann Emerg Med. 2023;81(4):468-481. doi:10.1016/j.annemergmed.2022.07.015 36117011

[31] Braun HM Walter C Farrell N Biello KB Taylor JL . HIV exposure prophylaxis delivery in a low-barrier substance use disorder bridge clinic during a local HIV outbreak at the onset of the COVID-19 pandemic. J Addict Med. 2022;16(6):678-683. doi:10.1097/ADM.0000000000000991 36383918 PMC9653062

[32] Karris MY Beekmann SE Mehta SR Anderson CM Polgreen PM . Are we prepped for preexposure prophylaxis (PrEP)? Provider opinions on the real-world use of PrEP in the United States and Canada. Clin Infect Dis. 2014;58(5):704-712. doi:10.1093/cid/cit796 24319083 PMC3922214

[33] Masson M Lamoureux J de Guise E . Self-reported risk-taking and sensation-seeking behavior predict helmet wear amongst Canadian ski and snowboard instructors. Can J Behav Sci. 2020;52(2):121-130. doi:10.1037/cbs0000153

[34] Thompson D Thompson R Rivara F . Risk compensation theory should be subject to systematic reviews of the scientific evidence. Inj Prev. 2001;7(2):86-88. doi:10.1136/ip.7.2.86 11428570 PMC1730707

[35] Marcus JL Glidden DV Mayer KH , et al. No evidence of sexual risk compensation in the iPrEx trial of daily oral HIV preexposure prophylaxis. PLoS One. 2013;8(12):e81997. doi:10.1371/journal.pone.0081997 PMC3867330 24367497

[36] Sagaon-Teyssier L Suzan-Monti M Demoulin B , et al. Uptake of PrEP and condom and sexual risk behavior among MSM during the ANRS IPERGAY trial. AIDS Care. 2016;28(suppl 1):S48-S55. doi:10.1080/09540121.2016.1146653 PMC4828609 26883400

[37] Ayerdi Aguirrebengoa O Vera García M Arias Ramírez D , et al. Low use of condom and high STI incidence among men who have sex with men in PrEP programs. PLoS One. 2021;16(2):e0245925. doi:10.1371/journal.pone.0245925 PMC7861516 33539363

[38] Chemtob D Weil C Hannink Attal J Hawila E Noff Sadeh E . HIV pre-exposure prophylaxis (PrEP) purchase patterns and STI occurrence among Israeli men: a cohort analysis. PLoS One. 2021;16(11):e0259168. doi:10.1371/journal.pone.0259168 PMC8601516 34793473

[39] McManus H Grulich AE Amin J , et al. Comparison of trends in rates of sexually transmitted infections before vs after initiation of HIV preexposure prophylaxis among men who have sex with men. JAMA Netw Open. 2020;3(12):e2030806. doi:10.1001/jamanetworkopen.2020.30806 PMC7758809 33355675

[40] Montaño MA Dombrowski JC Dasgupta S , et al. Differences in sexually transmitted infection risk comparing preexposure prophylaxis users and propensity score matched historical controls in a clinic setting. AIDS. 2019;33(11):1773-1780. doi:10.1097/QAD.0000000000002281 31149948 PMC8890685

[41] Morgan E Dyar C Newcomb ME D’Aquila RT Mustanski B . PrEP use and sexually transmitted infections are not associated longitudinally in a cohort study of young men who have sex with men and transgender women in Chicago. AIDS Behav. 2020;24(5):1334-1341. doi:10.1007/s10461-019-02664-9 31489520 PMC7056520

[42] Psomas CK Penaranda G Retornaz F , et al. A cohort analysis of sexually transmitted infections among different groups of men who have sex with men in the early era of HIV pre-exposure prophylaxis in France. J Virus Erad. 2022;8(1):100065. doi:10.1016/j.jve.2022.100065 35251684 PMC8891709

[43] Jenness SM Weiss KM Goodreau SM , et al. Incidence of gonorrhea and chlamydia following human immunodeficiency virus preexposure prophylaxis among men who have sex with men: a modeling study. Clin Infect Dis. 2017;65(5):712-718. doi:10.1093/cid/cix439 28505240 PMC5848234

[44] Centers for Disease Control and Prevention. Condom availability programs (CAPs). Published October 24, 2019. Accessed June 17, 2023. https://www.cdc.gov/healthyyouth/healthservices/caps/index.htm

[45] Myers JE Sepkowitz KA . A pill for HIV prevention: déjà vu all over again? Clin Infect Dis. 2013;56(11):1604-1612. doi:10.1093/cid/cit085 23408681

[46] Mayer KH Mimiaga MJ VanDerwarker R Goldhammer H Bradford JB . Fenway Community Health’s model of integrated, community-based LGBT care, education, and research. In: Meyer IH Northridge ME , eds. The Health of Sexual Minorities: Public Health Perspectives on Lesbian, Gay, Bisexual and Transgender Populations. Springer; 2007:693-715.

[47] Casanova-Perez R Apodaca C Bascom E , et al. Broken down by bias: healthcare biases experienced by BIPOC and LGBTQ+ patients. AMIA Annu Symp Proc. 2021;2021:275-284. 35308990 PMC8861755

[48] US Department of Health and Human Services. For providers. Published October 13, 2022. Accessed October 30, 2022. https://telehealth.hhs.gov/providers

[49] Nurx. PrEP. 2022. Accessed September 8, 2023. https://www.nurx.com/prep

[50] Mistr. About Mistr. 2022. Accessed September 8, 2023. https://heymistr.com

[51] Hamnvik OPR Agarwal S AhnAllen CG Goldman AL Reisner SL . Telemedicine and inequities in health care access: the example of transgender health. Transgend Health. 2022;7(2):113-116. doi:10.1089/trgh.2020.0122 36644516 PMC9829126

[52] Chiara A . The expanding role of pharmacists: a positive shift for health care. Commonwealth Medicine. Published March 26, 2019. Accessed September 8, 2023. http://hdl.handle.net/20.500.14038/27010

[53] National Alliance of State & Territorial AIDS Directors. Pharmacist-initiated PrEP and PEP. Published November 2021. Accessed September 8, 2023. https://nastad.org/sites/default/files/2021-11/PDF-Pharmacist-Initiated-PrEP-PEP.pdf

[54] California Pharmacists Association. SB 159 (Wiener) PrEP & PEP—pharmacists’ authority to furnish. Published October 8, 2019. Accessed October 30, 2022. https://cpha.com/wp-content/uploads/2019/10/PrEP-PEP-Fact-Sheet-10-8-2019.pdf

[55] Haberer JE Bangsberg DR Baeten JM , et al. Defining success with HIV pre-exposure prophylaxis: a prevention-effective adherence paradigm. AIDS. 2015;29(11):1277-1285. doi:10.1097/QAD.0000000000000647 26103095 PMC4480436

[56] Cohen RA Martinez ME Cha AE Terlizzi EP . Health insurance coverage: early release of estimates from the National Health Interview Survey, January–June 2021. Published November 16, 2021. Accessed October 13, 2022. https://www.cdc.gov/nchs/data/nhis/earlyrelease/insur202111.pdf

[57] Menon AA Nganga-Good C Martis M , et al. Linkage-to-care methods and rates in U.S. emergency department–based HIV testing programs—a systematic literature review brief report. Acad Emerg Med. 2016;23(7):835-842. doi:10.1111/acem.12987 27084781 PMC4938722

[58] Haukoos JS White DAE Lyons MS , et al. Operational methods of HIV testing in emergency departments: a systematic review. Ann Emerg Med. 2011;58(1 suppl 1):S96-S103. doi:10.1016/j.annemergmed.2011.03.017 PMC3690123 21684417

[59] Carlisle NA Booth JS Rodgers JB Heath SL Walter LA . Utilizing laboratory results to identify emergency department patients with indications for HIV pre-exposure prophylaxis. AIDS Patient Care STDS. 2022;36(8):285-290. doi:10.1089/apc.2022/0066 35951447

[60] Musoke L Allen KA Bray K , et al. Impact of a combined education and data driven intervention on PrEP uptake at the Veterans Health Administration. Open Forum Infect Dis. 2021;8(suppl 1):S514-S515. doi:10.1093/ofid/ofab466.1044

[61] Goedel WC Coats CS Chan PA , et al. A pilot study of a patient navigation intervention to improve HIV pre-exposure prophylaxis persistence among Black/African American men who have sex with men. J Acquir Immune Defic Syndr. 2022;90(3):276-282. doi:10.1097/QAI.0000000000002954 35312652 PMC9203974

[62] Burns PA Omondi AA Monger M , et al. Meet me where I am: an evaluation of an HIV patient navigation intervention to increase uptake of PrEP among Black men who have sex with men in the Deep South. J Racial Ethn Health Disparities. 2022;9(1):103-116. doi:10.1007/s40615-020-00933-1 33403654

